# Evaluation of cultural control and resistance‐breeding strategies for suppression of whitefly infestation of cassava at the landscape scale: a simulation modeling approach

**DOI:** 10.1002/ps.5816

**Published:** 2020-04-06

**Authors:** Hazel Parry, Andrew Kalyebi, Felix Bianchi, Peter Sseruwagi, John Colvin, Nancy Schellhorn, Sarina Macfadyen

**Affiliations:** ^1^ CSIRO, Ecoscience Precinct Brisbane Queensland Australia; ^2^ National Crops Resources Research Institute Kampala Uganda; ^3^ Farming Systems Ecology, Wageningen University and Research Wageningen The Netherlands; ^4^ Tanzania Agricultural Research Institute – Mikocheni Dar es Salaam Tanzania; ^5^ NRI, University of Greenwich Kent UK; ^6^ CSIRO, Black Mountain Canberra Australian Capital Territory Australia

**Keywords:** cassava mosaic disease, cassava brown streak virus disease, Leslie matrix model, whitefly‐transmitted viruses, spatial simulation, host‐plant resistance, cellular automata

## Abstract

**BACKGROUND:**

The whitefly *Bemisia tabaci* is an important vector of virus diseases, impacting cassava production in East Africa. To date, breeding efforts in this region have focused on disease resistance. Here we use a spatially‐explicit simulation model to explore how breeding strategies for whitefly resistance will influence the population dynamics of whitefly in the context of regional variation in cassava crop management practices.

**RESULTS:**

Simulations indicated that regions with a short cropping cycle and two cropping seasons per year were associated with high whitefly abundance. Nymph mortality and antixenosis resistance mechanisms were more effective than mechanisms that lead to longer whitefly development times. When spatial variation was introduced in heterogeneous landscapes, however, negative consequences of the antixenosis effect were observed in fields containing whitefly susceptible varieties, unless the proportion of whitefly resistant variety in the landscape was low (~10%) or the amount of matrix in the landscape was high (~75%).

**CONCLUSION:**

We show the importance of considering cropping regime and landscape management context when developing and deploying whitefly‐resistant cassava varieties. Recommendations differ significantly between regions. There may also be unintended negative consequences of higher whitefly densities for whitefly susceptible varieties if uptake of the new variety in a landscape is high, depending on the mechanism of resistance and the landscape context. Furthermore, we show that in some cases, such as where there is substantial fallow combined with a short single‐season crop, the management characteristics of the existing cropping regime alone may be effective at controlling whitefly populations. © 2020 The Authors. *Pest Management Science* published by John Wiley & Sons Ltd on behalf of Society of Chemical Industry.

## Graphical abstract

We show the importance of considering cropping regime and landscape management context when developing and deploying whitefly‐resistant cassava varieties. In some cases, management practice alone could substantially reduce the pest.

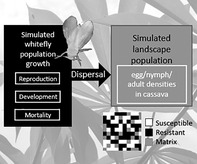

## 1 INTRODUCTION

Cassava is an important staple crop across sub‐Saharan Africa (SSA), where over half of global production is based.^1^ However, high population densities of *Bemisia tabaci* species now occur frequently in the region (in SSA, this is a pest complex of multiple *Bemisia tabaci* species^2^). Besides vectoring the viruses that cause cassava mosaic disease (CMD) and cassava brown streak disease (CBSD), additional yield loss due to *B. tabaci* can also occur from direct feeding damage and the deposition of sooty mould.^3^ The resultant annual regional cassava production loss due to the two diseases is estimated at more than $US 1 billion.^4^ There are limited chemical control options for *B. tabaci* species in cassava as insecticides are not accessible to many small‐holder farmers, and there is little knowledge of natural enemies.^5^ While alternative control methods exist, including cultural control and breeding for whitefly resistant cassava, these options have not been well explored, and knowledge on the effectiveness of these methods to suppress whitefly populations and how this may unfold in different landscape contexts is lacking.

Management tools to reduce losses due to pests and diseases should be considered a priority for crops such as cassava that contribute directly to food security for small‐holder farmers. A large research gap remains, and there is a need to consider cassava plant breeding aimed at incorporating resistance to whitefly in East African varieties. With limited resources to invest in breeding programs, a better understanding of the effects of the widely differing crop cultivation practices as well as a targeted approach to cassava breeding and the deployment of new varieties for whitefly resistance is advisable. Simulation modeling is a cost‐effective approach to explore the bewildering array of combinations of crop management, properties of new cassava varieties, and landscape contexts. Without such insight, short‐sighted and ineffective decisions could be made on the strategy to manage this pest and the type of whitefly resistance mechanism to select for (if any).

Whitefly population dynamics are, among other factors, governed by cassava availability and suitability, which in turn is influenced by crop cultivation regime. There is a wide diversity of cassava cultivars and management practices (e.g., planting dates and cropping periods) used by farmers in different regions. In Uganda, Malawi, and Tanzania, farmers plant cassava during the wet season when rains arrive, which is either during a 3‐month period in the first half of the calendar year, ‘Masika’, or in the second half of the year, ‘Vuli’. Cassava crops are grown across one or two planting seasons and remain in the ground for a period ranging from 8–18 months. The optimum cassava growing period based on yield optimization depends strongly on variety (i.e., there are both short‐ and long‐duration varieties). Long‐duration varieties are preferred when labor for harvesting is uncertain, or they mitigate the risk of food shortages at certain times of the year.

Theoretical studies have indicated that mobile pests can be suppressed by greater cropping synchrony across a landscape when this results in a period when host plants are not available in between crop cycles.^6^ Indeed, cassava cropping schedules and spatial arrangements that provide some spatial or temporal isolation have been associated with reduced disease pressure^7^ (augmenting management by clean‐cuttings). Furthermore, manipulation of cassava planting date has been identified as a potential practice to manage whitefly infestations; however, the full potential, practicability, and implications have not yet been studied.^7^ It is only in recent work that the relationship between cassava age (and associated host quality) and whitefly infestation level has been quantified.^8^ To date, key effects of cultivation practices on whitefly populations in cassava have not been investigated, including: (i) single versus double planting throughout the year, (ii) the time until harvest, (iii) the spatio‐temporal distribution of host plants in the landscape, and (iv) the interaction these practices may have with whitefly resistance traits in cassava.

Besides plant breeding to increase yield, most research and breeding efforts for African cassava have focused on disease resistance, specifically to CMD^9^, ^10^, ^11^, ^12^ and (with less success) CBSD.^13^ Cultivars have been developed that suppress disease to the extent that the viral DNA of *African Cassava Mosaic Virus Cameroon strain* (ACMV‐CM) is undetectable beyond 7 days post inoculation.^14^ However, resistance to *East African Cassava Mosaic Virus* (EACMV) is less effective in the same cultivars. The cultivation of whitefly resistant varieties of cassava has lagged far behind the cultivation of disease‐resistant varieties,^4^, ^15^ and it remains a ‘valuable, but still underexploited’ opportunity.^16^ Breeding for Host Plant Resistance (HPR) to pests has great potential as a low‐cost, sustainable solution to help manage pest and disease problems in resource‐poor farming regions such as sub‐Saharan Africa (SSA).^7^, ^15^, ^17^

In focusing on breeding for resistance to disease only, little attention has been paid to the resistance or tolerance that new varieties have to the disease vector so that resistance to whitefly is rare in cultivated crops^15^ (but see Ariyo *et al*.^18^). In fact, some improved disease‐resistant African cassava varieties now support greater whitefly colonization and population growth.^19^ In South America, there has been a program of breeding for cassava resistance to whitefly, but for a different pest species (*Aleurotrachelus socialis* Bondar) to that found on cassava in Africa.^15^ Varieties that have been bred for resistance to *A. socialis* have been tested for their resistance to East African *B. tabaci* species, with only the cassava variety MEcu 72 currently showing some promise in resistance to whitefly.^19^ Multiple traits can potentially be selected for in breeding for arthropod resistance,^20^ such as (i) reduced oviposition and repellence (here termed ‘antixenosis’); (ii) longer development period; (iii) higher nymph mortality. The evaluation of arthropod resistant crop varieties is generally conducted in laboratory or field trials at the plant or field scale, while the broader impact of the new crop variety on pest infestations in the surrounding crops in the landscape is generally overlooked. To develop, introduce and encourage adoption of a new variety successfully, however, that suppresses an insect pest in an agricultural system, a mechanistic understanding of the potential efficacy of the new variety in the context of existing management practices would be highly valuable (and can help to avoid unintended negative outcomes).

This paper explores the potential efficacy of the introduction of whitefly resistant cassava varieties by modeling the spatio‐temporal dynamics of whiteflies in cassava landscapes with various cassava cultivation practices and proportions of whitefly resistant cassava (vs. whitefly susceptible cassava or matrix), as well as varying uptake of the resistant variety. More specifically, we compare whitefly populations under cultivation practices of single versus double planting and different times until harvest (between 8 and 18 months), evident in different parts of the SSA region. We then consider the potential impacts of three different mechanisms of whitefly resistance on the landscape populations (both in fields planted to the new variety and also populations in fields with whitefly susceptible varieties nearby), which are: (i) reduced oviposition and repellence (here termed ‘antixenosis’); (ii) longer development period; (iii) higher nymph mortality. These traits, hypothetically, could result in quite different impacts on the population dynamics of the whitefly in space and time, depending on the management context and proportion of uptake of the new variety. For this purpose, we developed a novel spatial simulation model to simulate the whitefly population dynamics and test scenarios of new variety introductions under the different cultivation practices common in SSA. We also compared varying proportions of uptake of the new variety in these contexts. The model scenarios were designed to inform the best strategies for encouraging the adoption of new varieties by farmers as they become available in the future. We also give a theoretical estimate of the benefits in terms of reduction in whitefly densities that may be achieved, if adoption were to be successful.

## 2 MATERIALS AND METHODS

**Figure 1:**
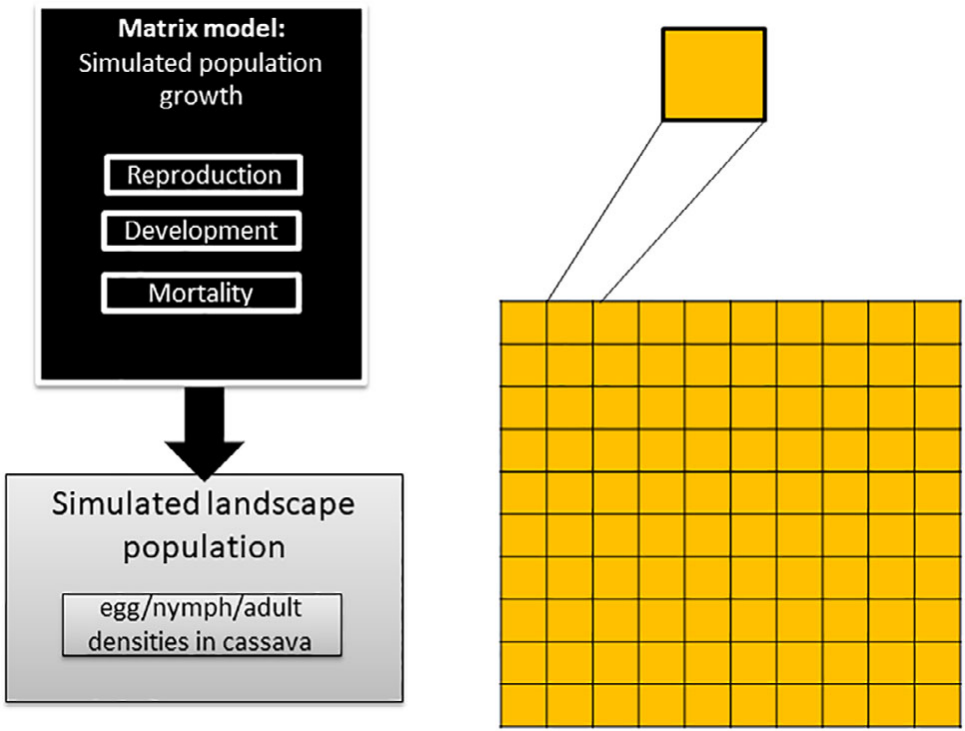
Overview of the model structure and the relationship between sub‐models.

The model comprises four sub‐models: (i) a cassava crop management model; (ii) a whitefly population dynamics model; (iii) a whitefly dispersal model; (iv) a cassava whitefly resistance model (Fig. 1). The model consists of a gridded simulation landscape, with crop ‘field’ cells being the ‘agents’ in the model, which were either cassava (whitefly resistant or whitefly susceptible) or unsuitable habitat for whiteflies (‘matrix’). The whitefly population within cells responds to the properties of the crop type grown in the field or matrix. As the cassava crop matures, it's quality as a whitefly host deteriorates, which has the effect of reduced population growth and increased dispersal. The model was written in Java, using the Repast Simphony 2.5 toolkit for agent‐based modeling.^21^

Within the fields, the model functions as a Leslie matrix model^22^ for the simulation of population dynamics of whiteflies, with a cellular automata approach used to simulate the dispersal of the adult whitefly population across the landscape. The landscape consists of 10 × 10 cells, with each cell measuring 10 × 10 m; this is comparable to the size of the fields observed in the three SSA case study regions presented (Andrew Hulthen, CSIRO, unpublished data). The model was run on a daily timestep. The whitefly population size in each cell of the landscape and the distribution of the population over time was the emergent result of the simulation model.

Within the case study regions, temperatures remain relatively constant throughout the year, and climatic effects (particularly rainfall) were accounted for indirectly by modeling the relationships between cassava age, cropping seasons and the whitefly population dynamics and dispersal. The assumption that temperature is not a driver of population dynamics behavior, as required in a Leslie matrix model, is therefore reasonable.

### 2.1 Cassava crop management model

**Figure 2:**
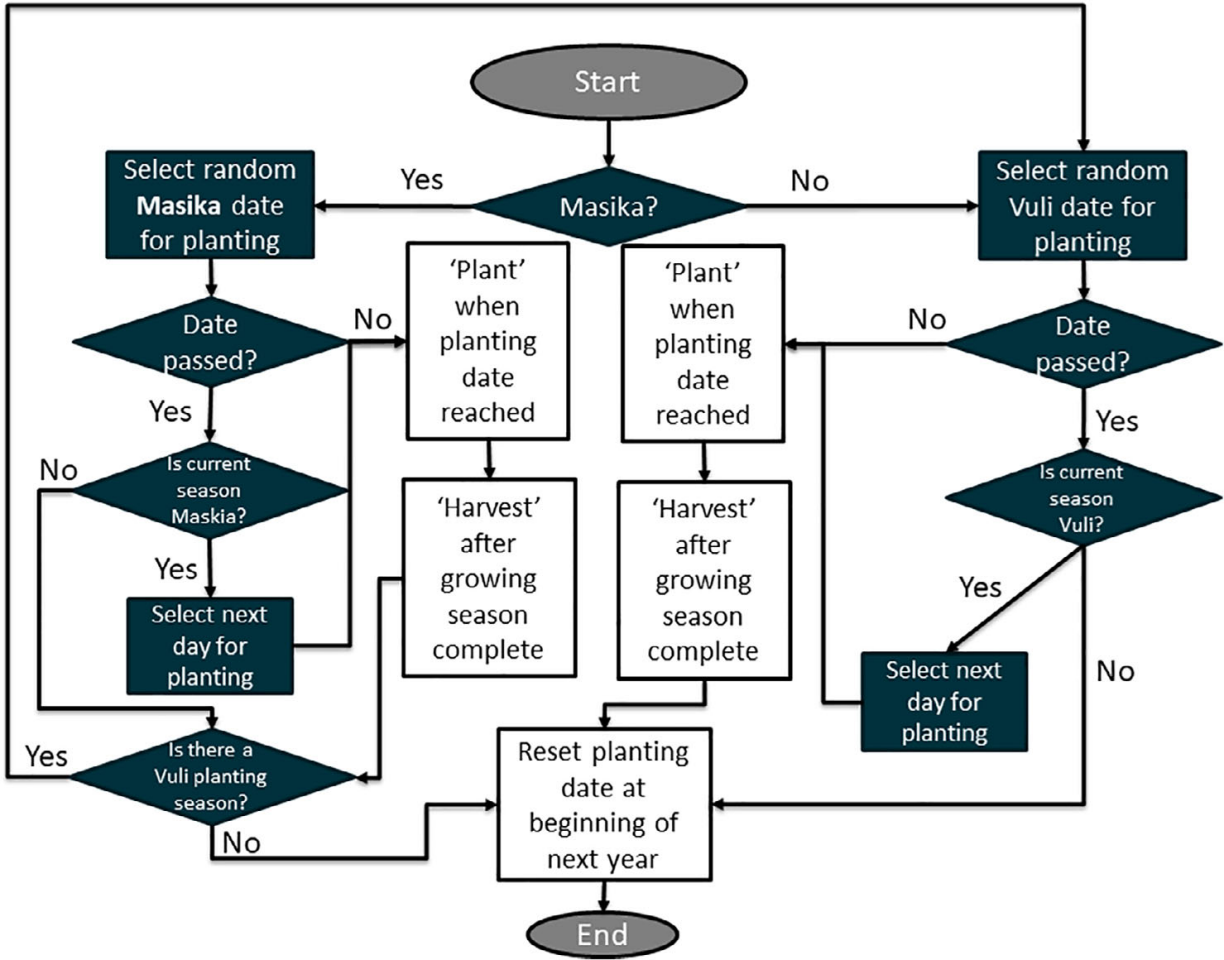
Flow diagram showing how the model decides when to plant for a two‐season model, starting at the beginning of each calendar year. For a single season, only the ‘Masika’ flow diagram applies.

The cassava crop cultivation regime for the whitefly population dynamics was modeled (Fig. 2), which allowed us to explore the emergent effects of varying cassava cultivation practices and traits on the whitefly population dynamics across the landscape. The model begins at the start of each calendar year by selecting at random whether to plant in the first (Masika) or second (Vuli) wet season, according to a proportion specified by the user. A random date within the user‐specified date range for these seasons is then chosen and set as the planting date. These are the only stochastic processes in an otherwise deterministic model. Once the crop is planted, the age of the crop is tracked as a state variable that influences the whitefly population dynamics (see below). Once the growing season is complete, the crop is harvested, and the properties of the landscape cell are reset. Whether the next cassava crop is planted straight‐away or if there is a fallow period will depend on the harvest date and the planting dates specified following the logic given in Fig. 2.

### 2.2 Population dynamics model

We used a stage‐structured Leslie matrix model^22^, ^23^ to simulate the whitefly population dynamics within each crop field cell. The model was structured in six development stages (egg, four nymphal stages, and adult). As the observed mean development times were approximately 6, 4, 3, 4, and 6 days for the five juvenile stages,^24^, ^25^, ^26^, ^27^ a Leslie matrix with two stages for eggs and two stages for the 5th nymph stage was created, leading to a total of eight stages. Calculations were made and stages transitioned every third day in the model (as there is a daily time step), so the seven immature stages translated to a development time of 21 days. This development time is close to empirically assessed development times of 22 days (in the lab)^28^ and 21 days (in the field),^29^ although this is at the lower boundary of development times observed for three *Bemisia tabaci* SSA taxa collected in East Africa.^30^ The adults survived for only 3 days^31^ based on the whitefly species MEAM1, although this may be an underestimate.

The reproduction rate of whitefly females averages four eggs per day,^28^, ^31^, ^32^ with the simulated fecundity therefore 12 eggs over the three days of adulthood. This fecundity was somewhat low but comparable to the mean fecundity given in the literature: a mean of 8 (range 1–41) eggs per female (MEAM1)^31^ and 14–49 eggs per female, dependent on the variety of cassava.^33^ The survival under the highest suitability was set at 95%, which is within the range of survival found across life stages in the literature: 73%–95% (N4^34^ ‐ Eggs^32^).

Density‐dependent survival (DD) was calculated as follows with the parameters *k* (strength of density dependence, see Table 2) and *N* (the total population per plant) and implemented as a multiplier of the daily reproduction rate (Table 1).

(1) $$\mathrm{DD}=\frac{1}{1+\mathrm{kN}}$$

**Table 1:** Baseline parameterization of whitefly model (without resistant variety or aging effects)

Parameter	Value	Egg	N1	N2	N3	N4	Adult	Unit	Reference
Survival rate^a^^,^ b		0.8	0.95	0.95	0.95	0.95	0.8	Per stage	32, 34
Development time per stage^a^		6	3	3	3	6	‐	days	24, 25, 26, 27
Reproduction rate	4							Eggs/fem/day	28, 31, 32
Adult survival	3							days	31

a Varies depending on the scenario for whitefly resistant variety.

b Varies with cassava aging effect.

**Figure 3:**
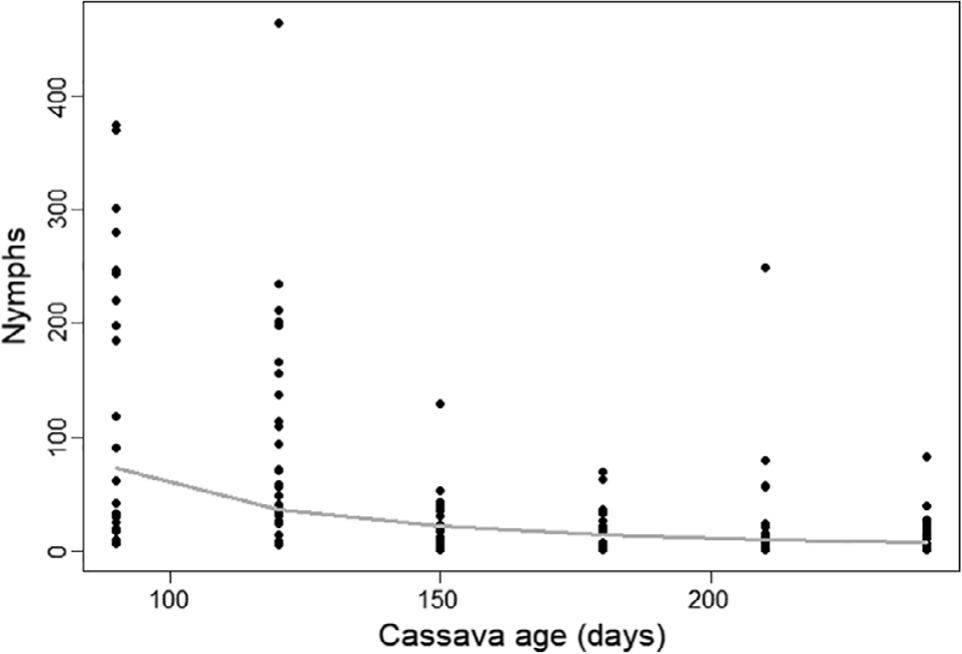
Relationship between whitefly nymph populations and the cassava age, F_(1,158)_ = 70.37, R‐sq = 0.308.

Young cassava is highly suitable for whitefly, so the strength of density dependence on whitefly population growth was low (*k* = 3). This parameterization reflected the population metrics of R_0_ = 1.56 and r_max_ = 0.014 d^−1^, which is in line with metrics for whitefly development on cassava.^31^ The age of the cassava, which determines the host plant quality, had an influence on the simulated population dynamics in terms of increased dispersal (see below) and decreased ‘suitability’ of the crop.^35^, ^36^ The decline in crop suitability from 3 (high) to the minimum suitability of 1.5 (low) was calculated daily once the cassava reached 80 days old, by multiplying the current suitability by a fraction p (suitability). The function was derived from data^8^ that indicated a log–log relationship between whitefly nymph population abundance and the cassava age (Fig. 3): this relationship was scaled to a proportion between 0 and 1 to estimate the proportion by which to multiply a maximum suitability index value of 3 each daily timestep:

(2) $$P\left(\text{suitability}\right)=\frac{e^{15.026-\left(2.389\ln\left(\mathrm{age}-1\right)\right)}}{100}$$

Based on this suitability index (integer values between 1 and 3), cassava crop suitability as a function of age determined the parameters used in the matrix model, which represented the potential for whitefly population growth (relative growth rate), see Table 2.

**Table 2:** The values for the parameters k (strength of density dependence) and survival per life stage under ‘high’, ‘medium’, and ‘low’ suitability of the crop

Parameter: Suitability scenario:	k (strength of density dependence)	Survival per stage (all life stages)
High suitability (3)	3	As per Table 1
Medium suitability (2–3)	5	0.8
Low suitability (1–2)	7	0.7

### 2.3 Dispersal model

The population adaptation to the habitat within each cell and the age of the cassava crop was based on the population acting to maximize fitness (foraging for younger plants), with consequences for increased reproduction and persistence of the population. Whiteflies tend to move from mature cassava plants and other host‐plants in the surrounding landscape to colonize young cassava crops whose foliage is more succulent and palatable.^37^, ^38^, ^39^ Young cassava is more palatable during the first few months as it is producing leafy growth in that time, with tuberous roots developing later – this affects the nutritional quality of the plant and hence the dynamics and activity of whitefly.^39^

We assumed that the relative departure rate of adult whiteflies was dependent on the host‐plant quality (cassava age) and that if dispersal occurs, some proportion of the adult population will emigrate (with no dispersal when the crop is <80 days old). The proportion of whiteflies remaining (i.e., not dispersing) was reduced each daily timestep after 80 days to account for the effect of the age of the cassava, in the same manner as the suitability of the cassava (see Eq. 2). A von Neumann transfer (i.e., to four neighboring cells) of the dispersing adult population was applied to move the dispersing population equally into the surrounding cells, for each daily timestep.

### 2.4 Cassava whitefly resistance model

The three main mechanisms by which cassava may exhibit resistance to whitefly are: (i) reduced oviposition and repellence (termed ‘antixenosis’); (ii) longer whitefly development period; and (iii) higher nymph mortality (Table 3).

**Table 3:** Mechanism of cassava whitefly resistance and method to implement in the model based on data from Bellotti and Arias^15^

Resistance mechanism	Model values	Data
Antixenosis: reduced oviposition (due to repellence)	Antixenosis (25% increased relative departure rate (including from young plants), 25% reduced reproduction rate)	*Aleurotrachelus socialis* on cassava (lowest oviposition rate variety 18% of highest rate)
Longer development period	34.5 days (an increase of 2 days per 3‐day stage interval was applied across the lifestages)	*Aleurotrachelus socialis* on cassava with M ECU 72 variety
Higher nymph mortality	72.5% (i.e., only 27.5% survival) across all nymph stages	*Aleurotrachelus socialis* on cassava with M ECU 72 variety

### 2.5 Scenarios

#### 2.5.1 *Crop cultivation regime*

We first considered the population dynamics of whitefly in response to the cassava cultivation regime over time (double vs. single planting and time to harvest, Table 4), on a null landscape comprising 100% whitefly susceptible cassava.

**Table 4:** Theoretical cropping scenarios explored with the model, based on existing management practice

**Seasonality of management**	Single season			Double season		
**Crop cycle length (months)**	8	10	12	14	16	18
**Percentage of whitefly resistant cassava in the landscape**	10%		50%		70%	
**Percentage of ‘matrix’ in the landscape**	‘low’ (25%)			‘high’ (75%)		

#### 2.5.2 *Crop cultivation regime – comparing different regions of SSA*

We then parameterized the model based on regional management practices in Uganda, Tanzania, and Malawi, to allow us to draw some ‘real‐world’ conclusions on the effects of cultivation regime (Table 5, see also Supporting Information Fig. S1 for map).

**Table 5:** Sub‐Saharan Africa case study regions cropping scenarios explored with the model

**Uganda**	Kamuli	Lira	Rakai
1st rain timing	Apr–Jun (91–181)	Mar–Jun (60–181)	Apr–May (91–151)
2nd rain timing	Aug‐Oct (213–304)	Aug‐Oct (213–304)	Aug‐Oct (213–304) (rare)
% Cassava planted at first rain	60	60	80
Cassava duration (days)	365–425		
% uptake of the new variety	70		
**Tanzania**	Dar es Salaam/Bagamoyo	Dodoma	Mwanza
1st rain timing	Mar–May (60–151)	Dec‐Mar (1–90)	Feb‐May (32–151)
2nd rain timing	Oct‐Dec (274–365)	–	Sept‐Dec (244–365)
% Cassava planted at first rain	60	100	50
Cassava duration (days)	305–365		
% uptake of the new variety	10		
**Malawi**	Lilongwe	Karonga	
1st rain timing	Dec‐Jan (1–60)	Jan‐Feb (1–60)	
2nd rain timing	–	–	
% Cassava planted at first rain	100	100	
Cassava duration (days) (fallow possible)	210–270	300–365	
% uptake of the new variety	50		

#### 2.5.3 *Crop cultivation regime and different mechanisms of whitefly resistance*

We implemented three scenarios of whitefly resistance mechanisms in cassava (Table 3) and explored what the impact on population dynamics of the whitefly would be in the null landscape over time, given the different scenarios of a crop‐cultivation regime (as above). We compare these to a null model: i.e., a reference scenario of no resistance to whitefly in cassava.

#### 2.5.4 *Different mechanisms of whitefly resistance with different uptake of resistant varieties*

A more realistic scenario is when uptake of resistant varieties across a landscape is not 100%, so the landscape is a mix of whitefly resistant and whitefly susceptible varieties. Thus, we were also interested in the effects of contrasting proportions of whitefly resistant cassava in the landscape, representing variation in the uptake of new varieties. We then used further scenarios to explore the theoretical effects of varying management practices on the whitefly population dynamics in mixed landscapes, both within fields containing the whitefly resistant cassava variety and within fields of a whitefly susceptible variety in the landscape. We created three landscapes: (i) high uptake (70% whitefly resistant cassava); (ii) medium uptake (50% whitefly resistant cassava); and (iii) low uptake (10% whitefly resistant cassava) (Table 4 and Supporting Information Fig. S2). These proportions of uptake corresponded to uptake observed for disease‐resistant varieties in Uganda, Malawi, and Tanzania, which were high, medium, and low, respectively.^40^ The scenarios considered the effects of adult movement with the whitefly dispersal model and explored the effects of different mechanisms of whitefly resistance in cassava with the cassava whitefly resistance model.

#### 2.5.5 *‘Smallholder’ versus ‘commercial’ landscapes*

We then also explored the effects of varying the percentage uptake of the new variety and the percentage ‘matrix’ (unsuitable habitat for whitefly) in the landscape. We characterized a ‘smallholder’ landscape as one that has a high percentage of ‘matrix’ habitat (75%). We assumed the ‘matrix’ was not suitable for whitefly based on available evidence of crop/non‐crop hosts in the region besides cassava.^4^, ^37^, ^41^, ^42^, ^43^, ^44^ A ‘commercial’ landscape had a lower percentage of ‘matrix’ (25%). We also explored the effect of the matrix and so created two additional landscapes with the 50% uptake scenario: one with 25% matrix and the other with 75% matrix (see Supporting Information Fig. S2).

#### 2.5.6 *Crop cultivation regime and different mechanisms of whitefly resistance – comparing different regions of SSA (with varying uptake of the resistant variety)*

Finally, we combined the whitefly resistance scenarios (Table 3) with the regional management practice scenarios (Table 5), to explore the likely outcomes of breeding for different whitefly resistance mechanisms in cassava varieties in each region. This incorporated estimated differences in uptake by region, and therefore the proportion of the whitefly resistant variety in the landscape. These scenarios allowed an assessment of what mechanism for whitefly resistance, if any, would be most advantageous to cultivate in each given region.

The model output was summarized across all field agents of the same type (i.e., cassava, whitefly resistant cassava, or matrix). The output data collected daily was the number of eggs, nymphs, and adults within each field as well as the field type and management applied. The model was initialized with a low initial density of 0.0125 per m^2^ adult whitefly in all cells of the landscape, and ‘background immigration’ of whitefly occurred again in the model at this rate if the adult population dropped below this density during the simulation run. The model was run for 20 years for five replicate runs. To eliminate transient dynamics, we discarded output from the initial year in which the model stabilizes and used the results from the following 19 years.

### 2.6 Results

#### 2.6.1 *Crop cultivation regime*

**Figure 4:**
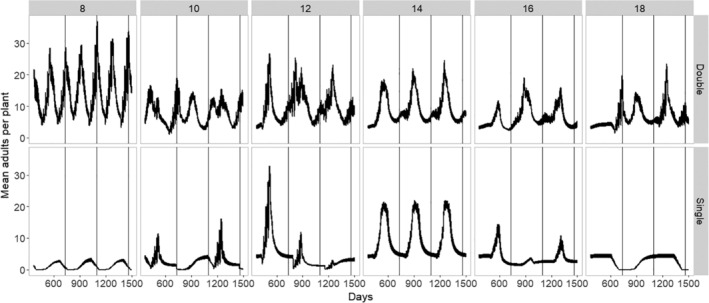
Simulated impact of management practice (time until harvest and double vs. single planting) on the adult whitefly population dynamics over time in the simulation (five replicate model runs).

The simulation output indicated that both the time until the harvest of the cassava crop and the planting schedules influenced both the abundance and the stability of the population (mean adults per plant) over time (Fig. 4). Whitefly populations in cassava were highest for double plantings, particularly when the time to harvest was short (8 months) due to the constant presence of young cassava in the landscape. When there was only a single planting, however, a short time to harvest did not result in high pest populations; here, the lowest mean populations during a single planting regime occurred when the time to harvest was either short or long. With a short time to harvest, there was increased fallow and a synchronized planting of the single crop; with a long time to harvest, there was decreased suitability due to the cassava age. In the single planting regime, whitefly populations were highest where there was the greatest overlap between whitefly populations; this was when the time until the harvest was 14 months (Fig. 4).

### 2.7 Crop cultivation regime – comparing different regions of SSA

**Figure 5:**
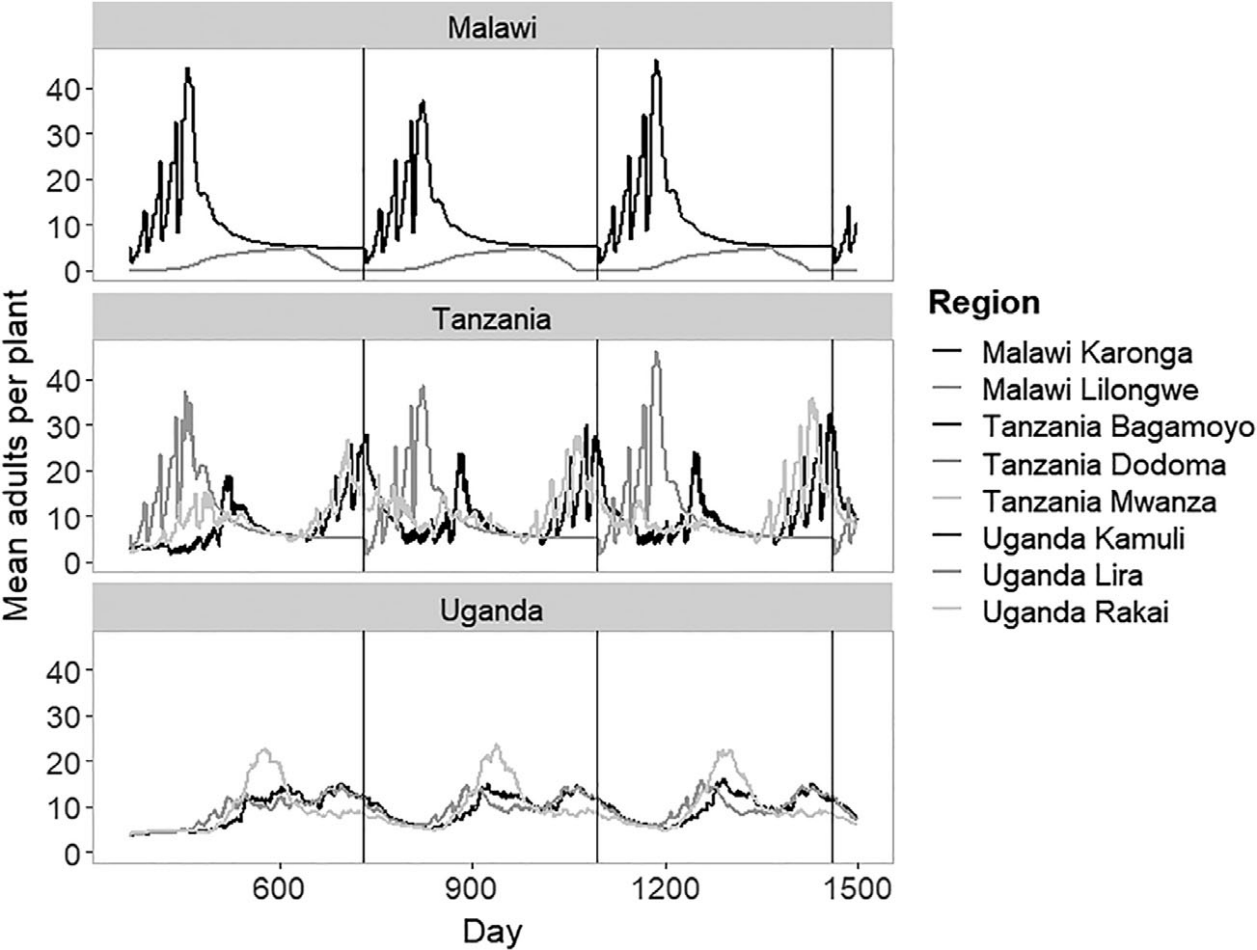
Simulated impact of management practice (time until harvest and double vs. single planting) on the adult whitefly population dynamics over time in regions of Malawi, Tanzania, and Uganda. See Table 5 for the characteristics of each region.

There were substantial differences in timing and planting schedules between the cropping practices in each region (Table 5). The differences in whitefly populations explored with theoretical cropping regimes, therefore, were reflected in the regional cropping whitefly population differences (Fig. 5). Lilongwe (Malawi), which had a short single cropping season with fallow periods, stood out as having very low expected whitefly populations. The regions of Karonga in Malawi and Dodoma in Tanzania also had a single cropping season, but it was longer, resulting in higher populations and the boom‐bust dynamics observed in the theoretical scenarios when the cycle is approximately annual. The other regions (Lilongwe in Malawi, Mwanza, and Bagamoyo in Tanzania and all regions in Uganda) have double‐cropping regimes of varying lengths (Table 5), which resulted in less variable population dynamics and similar mean adult populations per plant over time. The timing of the population peaks varied by region, according to the variation between regions in the planting windows; Tanzania and Malawi are similar, but Uganda has a different population cycle over time due to these differences in planting windows.

### 2.8 Crop cultivation regime and different mechanisms of whitefly resistance

**Figure 6:**
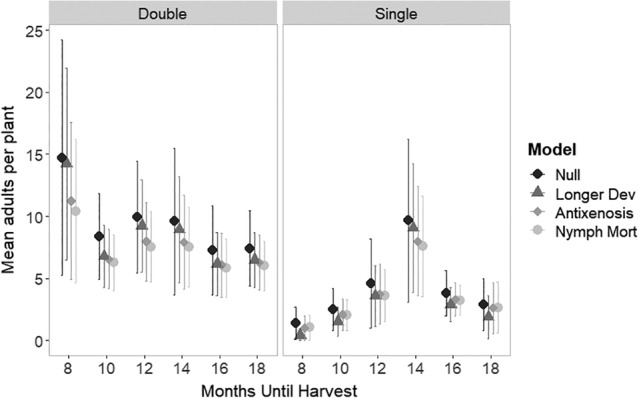
Simulated impact of the effects of different whitefly resistance mechanisms combined with management practice (Months until harvest and double vs. single planting) on the mean adult whitefly population in a single cassava field. Error bars show standard deviation over the 20‐year simulation period.

The mean adult population dynamics per plant averaged over a 20‐year simulation run (Fig. 6) without cassava improvement for whitefly resistance, as per Fig. 4, was summarized as the ‘null’ model. Differences between whitefly resistance scenarios (Table 3) were compared (Fig. 6). Improvement for whitefly resistance appeared to have the most impact on the highest whitefly populations (the double planting with short cropping cycle). Nymph mortality and antixenosis, which acted similarly in the case of a homogenous whitefly resistant crop landscape, appeared to be generally more effective at suppressing populations in the resistant crop than longer development time (Fig. 6). The exception to this was for the single planting cases with low populations (either short or long times until harvest, i.e., little overlap in populations), but in these cases, there was little impact of any whitefly resistant varieties overall and little difference between the resistance types.

### 2.9 Different mechanisms of whitefly resistance with different uptake of the resistant variety

**Figure 7:**
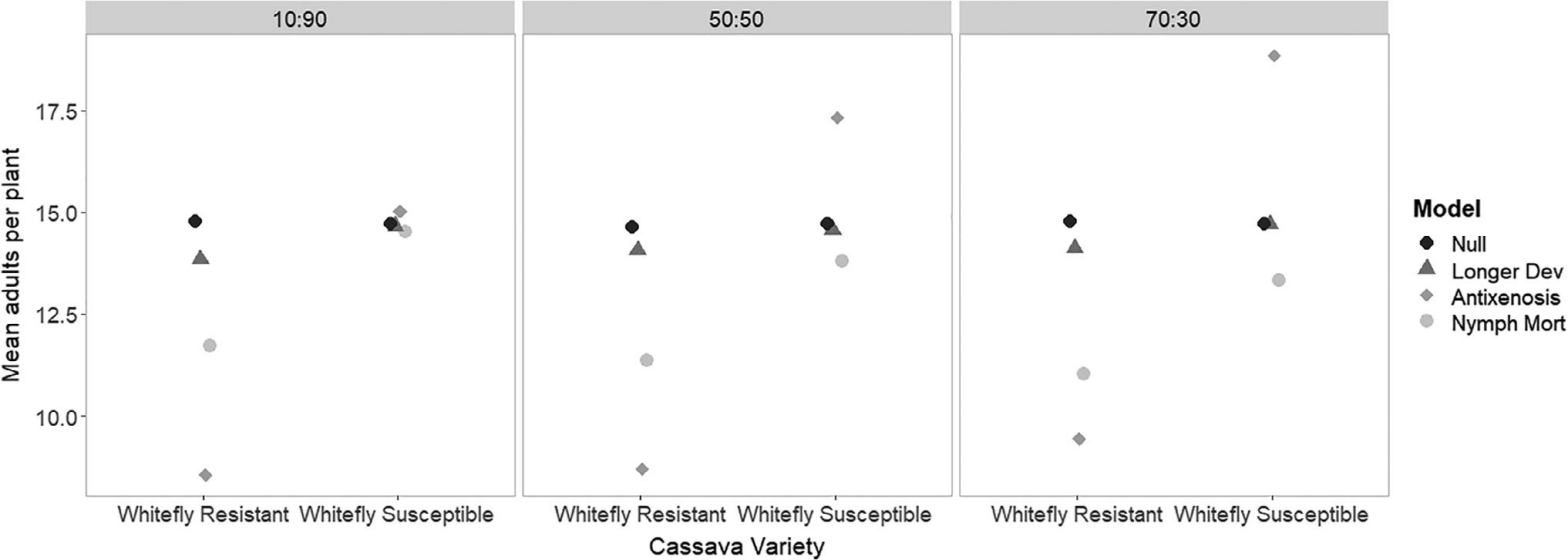
Simulated impact of the effects of different whitefly resistance mechanisms on the mean adult whitefly landscape population in the whitefly resistant and the whitefly susceptible cassava variety fields. Comparison of different ratios of whitefly‐resistant to whitefly‐susceptible varieties in the landscape.

Here, we compare the mean adults per plant under different scenarios of uptake (percentage of the new variety in the landscape) for both the fields growing the whitefly resistant variety and the whitefly susceptible variety (Fig. 7).

In all cases, the antixenosis whitefly resistance appeared to be the most effective at suppressing populations in the resistant crop, as in this scenario the whiteflies were moving away into the whitefly susceptible (and thus non‐repellent) variety crop resulting in large reductions in the population in the whitefly resistant crop fields. However, unless the uptake across the landscape was very low (10:90 whitefly resistant: whitefly susceptible), then there was likely to be a substantial negative effect of this repellence in fields that are growing whitefly susceptible varieties – i.e., an increased mean population of adults per plant. This effect appeared to increase with an increased proportion of the whitefly resistant variety in the landscape. However, the scenario of improvement with increased nymph mortality resulted in increased pest suppression with an increased proportion of uptake in the landscape, both within the fields growing the whitefly resistant variety and also in the whitefly susceptible crop fields.

### 2.10 ‘Smallholder’ versus ‘commercial’ landscapes

**Figure 8:**
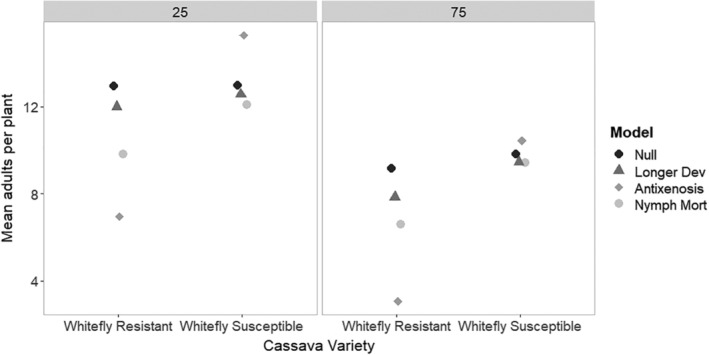
Simulated impact of the effects of different whitefly resistance mechanisms on the mean adult whitefly landscape population in the whitefly resistant and the whitefly susceptible cassava variety fields. Comparison of different proportions of ‘matrix’ habitat in the landscape (25% = ‘commercial’; 75% = ‘smallholder’).

Further exploring more realistic scenarios, we compared whitefly dynamics in landscapes that contained varying proportions of the matrix in the landscape, using the 50:50 scenario of whitefly resistant: whitefly susceptible varieties in the landscape under a double planting and short time to harvest scenario. We compared a low (25%) with a high (75%) proportion of matrix (representing a ‘commercial’ versus a ‘smallholder’ landscape, respectively; Fig. 8). We found that estimated populations were likely to be higher in the landscape with a low proportion of matrix (i.e., commercial), which is logical as there was more cassava habitat in which the whitefly populations could develop and into which they could disperse. We found similar effectiveness of the whitefly resistant cassava, with antixenosis again being most effective within the resistant crop, followed by nymph mortality. However, there was a greater negative impact on the whitefly susceptible variety crop fields (i.e., increased mean adult numbers per plant) when there was a low proportion of matrix (i.e., a ‘commercial’ landscape); there was much less negative impact when the proportion of matrix was high.

### 2.11 Crop cultivation regime and different mechanisms of whitefly resistance – comparing different regions of SSA (with varying uptake of the resistant variety)

**Figure 9:**
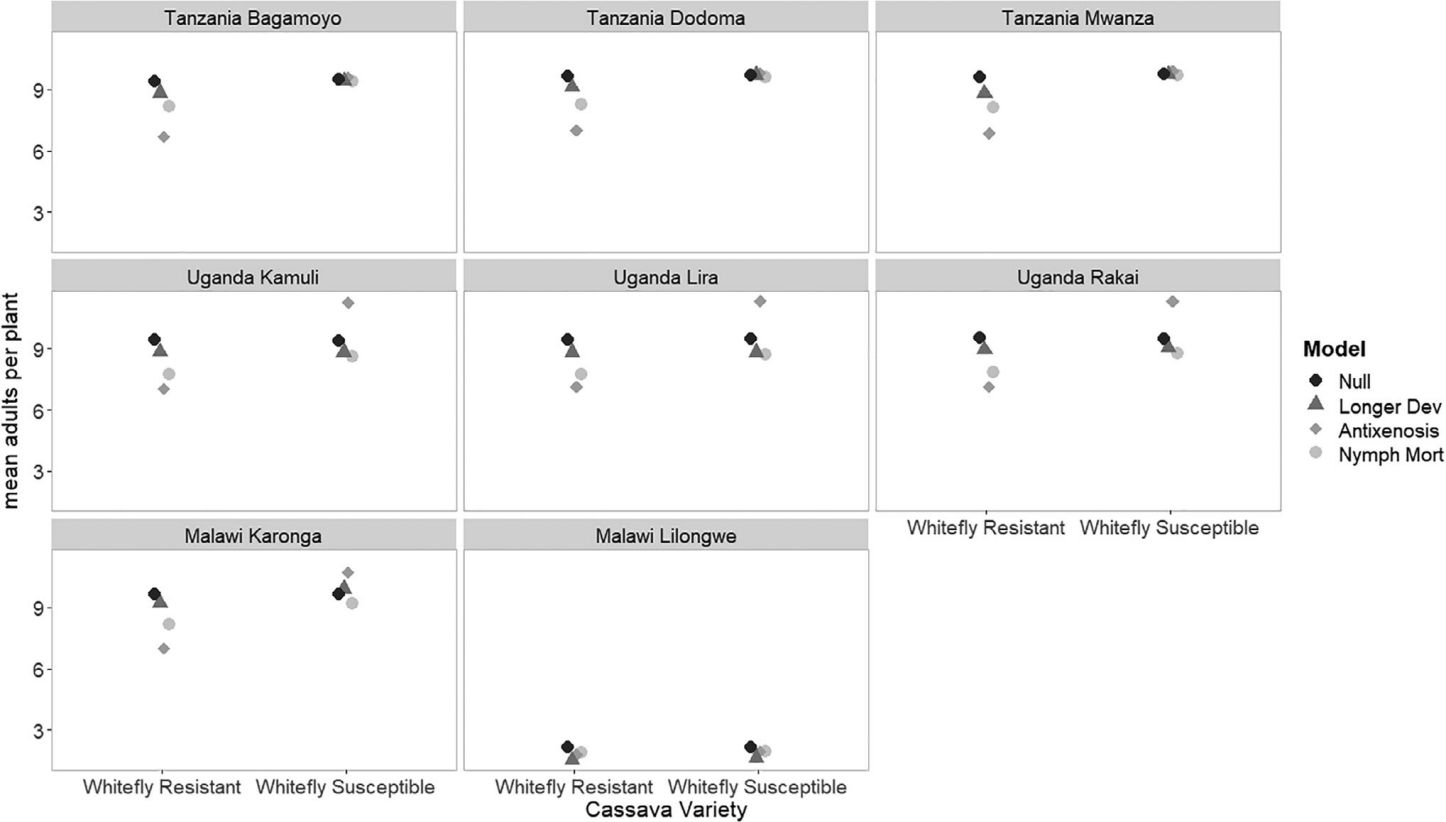
Simulated impact of the effects of different whitefly resistance mechanisms on the mean adult whitefly landscape population in the whitefly resistant and the whitefly susceptible cassava variety fields. Comparison of different regions.

Although the variation in whitefly populations over time differs between regions (Fig. 5), the mean adults per plant over 20 years in the null model (a whitefly susceptible variety) is very similar between regions, except for Lilongwe in Malawi (Fig. 9). There is little difference within countries in the scenario outcomes, except between the two regions in Malawi (Fig. 9). In Tanzania, the whitefly resistant variety with antixenosis was most effective at suppressing populations in the resistant crops with little negative consequence for whitefly susceptible varieties, due to the low uptake scenario of the whitefly resistant variety in this country (10%). However, Uganda was estimated to have a much higher uptake (70%), and thus there were likely to be negative consequences in terms of higher adult numbers per plant in the whitefly susceptible varieties if antixenosis is the whitefly resistance mechanism. Nymph mortality was also effective in the crop fields with whitefly resistant varieties in Uganda and did not have negative consequences for whitefly susceptible variety crop fields. Malawi Karonga was like Uganda, with negative consequences for whitefly susceptible variety crop fields when antixenosis was the mechanism of whitefly resistance in the crop fields. However, the effect was not particularly strong, as the uptake of the new variety in this region was estimated to be lower (50%). The low populations estimated because of fallow in the management practices in Malawi Lilongwe mean that varieties improved for whitefly resistance are unlikely to be of value to introduce.

## 3 DISCUSSION

The spatial simulation model incorporating the regional differences in management practices for cassava in Sub‐Saharan Africa (SSA) was used to investigate which whitefly resistance mechanisms would be most effective in suppressing whitefly populations across agricultural landscapes. Models are often used to evaluate the potential of different management actions, including to evaluate different disease or pest suppression strategies. We have shown here that the recommendations for management with whitefly resistant varieties of cassava vary immensely depending on the landscape context of that management action. Although several models have been developed that simulate disease dynamics,^45^, ^46^, ^47^, ^48^, ^49^ including resistance,^50^ models rarely consider the vector whitefly population dynamics and dispersal,^51^, ^52^, ^53^ particularly at the landscape scale, as we have done.

The model showed that double planting seasons in a year would lead to higher whitefly populations compared to a single planting season, due to greater overlap between crops and constant cultivation somewhere in the landscape, even when there was a short cropping cycle. This effect was due to the continuous availability of relatively young cassava in the landscape. In both the single and the double planting cases, overlap also appeared to result in a greater variance (boom and bust cycles) compared to regimes with fallow. These theoretical differences drive the variation in population dynamics seen when we parameterized the model to represent the management regimes of two to three regions in each of Malawi, Tanzania, and Uganda.

When introducing new varieties with various mechanisms of resistance, in the context of the cassava management practices, the model indicated that the spatial context of the amount of cassava in the landscape, the amount of matrix, and the cassava management practices all influenced what mechanism of whitefly resistance would likely be the most advantageous to breed into a new variety. Nymph mortality and antixenosis were generally more effective within the resistant crops than longer development time and acted similarly when the landscape was simulated with homogenous fields of the whitefly resistant variety. When more realistic spatial variation was introduced, however, we observed negative consequences of the antixenosis effect for fields containing whitefly susceptible varieties. The negative consequences were due to the repellence of the whiteflies from the whitefly resistant crops, which will increase populations in the whitefly susceptible crop fields unless the proportion of whitefly resistant variety in the landscape is very low (~10%) or the amount of matrix in the landscape is high (~75%), equivalent to something like a smallholder landscape.

From this theoretical exploration, we were then able to parameterize the model to represent our case study regions in SSA and draw the following conclusions on what mechanism for whitefly resistance would be most advantageous in each region. We conclude that the whitefly resistance trait may not be of any advantage in the Lilongwe region of Malawi, due to the overriding effect of management in suppressing whitefly populations (short single‐season crop cycles with fallow periods). In the Tanzanian regions with single or double cropping periods that were only likely to have a very low uptake of the new variety based on the current uptake of approximately 10% for disease‐resistant varieties (Bagamoyo, Dodoma, and Mwanza),^40^ the mechanism of antixenosis was likely to work well overall. We expect antixenosis would work well in these Tanzanian regions because it was most effective in reducing populations in the whitefly resistant fields without discernible negative consequences for the whitefly susceptible crop fields with that low level of uptake. However, when uptake is likely to be much higher under similar cropping regimes (regions in Uganda at ~70% and Karonga in Malawi at ~50%), then our results indicate that it may be advisable to breed for traits that confer nymph mortality, for effective landscape management of whitefly as a whole. This finding is of importance, as the cassava genotype Ecu72 resistance works in this manner. In all regions, these results may be affected by the proportion of matrix in the landscape (i.e., the intensity of cassava cropping), with an increased proportion of matrix likely to both reduce whitefly populations, but also to reduce negative consequences for whitefly susceptible variety fields.

The null model (whitefly susceptible varieties in each region) resulted in a similar mean whitefly abundance over time for each region due to the matrix modeling approach taken. In reality, there are climatic differences between the regions, which have been shown to impact on population size (see Supplementary Information Fig. S3); Malawi is the least climatically suitable for whitefly and Uganda the most suitable (when between‐season variation in weather is averaged out over a long historical time period). In this case, the cassava management practices in Malawi (particularly in the Lilongwe region where there is a single short cropping season with fallow periods) are likely to reinforce the climatic advantage the region has in low whitefly numbers. Conversely, the practices in Uganda and some regions of Tanzania (double planting with relatively short cycles) are likely to reinforce the environmental conditions for higher whitefly numbers. In all cases, the intensity of cultivation (i.e. the proportion of matrix) will have an influence, and this can vary greatly between landscapes.

This study indicates the importance of considering the spatial cropping regime and management context when developing new varieties for whitefly resistance. The mobility of the pest and mechanism of resistance that results in repellence can combine to have negative consequences for whitefly susceptible varieties in the landscape if uptake of the new variety in the landscape is high. However, if uptake of the new variety in a region is low, or the crop is surrounded by a high proportion of inhospitable matrix, then breeding for antixenosis could be very effective. In some cases, it may not be worth investing in deploying a whitefly resistant variety at all (although disease resistance is another matter), as the cropping regime may in itself be effective at controlling whitefly populations; in particular, if either a single long cropping season or a very short one with periods of fallow across the landscape. In these regions, other management options such as cultural control practices to disrupt the movement and oviposition of whitefly on new cassava plants,^54^ as well as the conservation of natural enemies, may be relatively more important. Thus, in general, our results indicate that breeders should consider diverse sources of resistance in breeding programs. The context in which the new varieties will be released, and the extent of uptake, will be an important consideration in deciding which mechanism is likely to be most effective. This model, therefore, provides a novel tool that is useful to scale‐up what is known about population dynamics of whitefly in relation to the age of cassava and to consider management practice effects at the landscape scale.

Concerning the introduction of whitefly resistant varieties, the next questions we could address with the model might be: when and where is it best to introduce a new variety to achieve significant landscape‐wide reductions in whitefly numbers? Could strategic introductions of particular resistance mechanisms be more effective than *ad‐hoc* deployments? Would spatial concentrations of new varieties be more effective at pest suppression with fewer negative impacts on whitefly susceptible varieties in the landscape? Similar questions for spatial planning for the release of transgenic insects into a landscape are currently being asked using empirical approaches,^55^ and simulation modeling might play a complementary role to assess the potential efficacy of different implementation strategies before making a significant investment in developing new crop varieties or genetically modified organisms. Furthermore, such scenarios can be used by policy‐makers and agricultural extension workers to inform the best use of limited resources for the multiplication and distribution of new planting material to farmers.^47^, ^49^

## Supporting information


**Figure S1**: Map of the Sub‐Saharan study region showing areas that were compared in terms of Cassava management regime in this study.
**Figure S2**: Artificial landscape scenarios used as model input in this study
**Figure S3**: Observed presence of whitefly adults per plant versus the environmental Index (EI) calculated using a climatic niche model^30^
