# Supplementary material for: Evaluation of cultural control and resistance‐breeding strategies for suppression of whitefly infestation of cassava at the landscape scale: a simulation modeling approach
Source: Pest Manag Sci. 2020 Apr 6;76(8):2699–710. doi: 10.1002/ps.5816 (PMC7383508; doi:10.1002/ps.5816)
Supplement: Supplementary file 1 — Figure S1: Map of the Sub‐Saharan study region showing areas that were compared in terms of Cassava management regime in this study. Figure S2: Artificial landscape scenarios used as model input in this study Figure S3: Observed presence of whitefly adults per plant versus the environmental Index (EI) calculated using a climatic niche model30 [file PS-76-2699-s001.docx]

# Supporting information


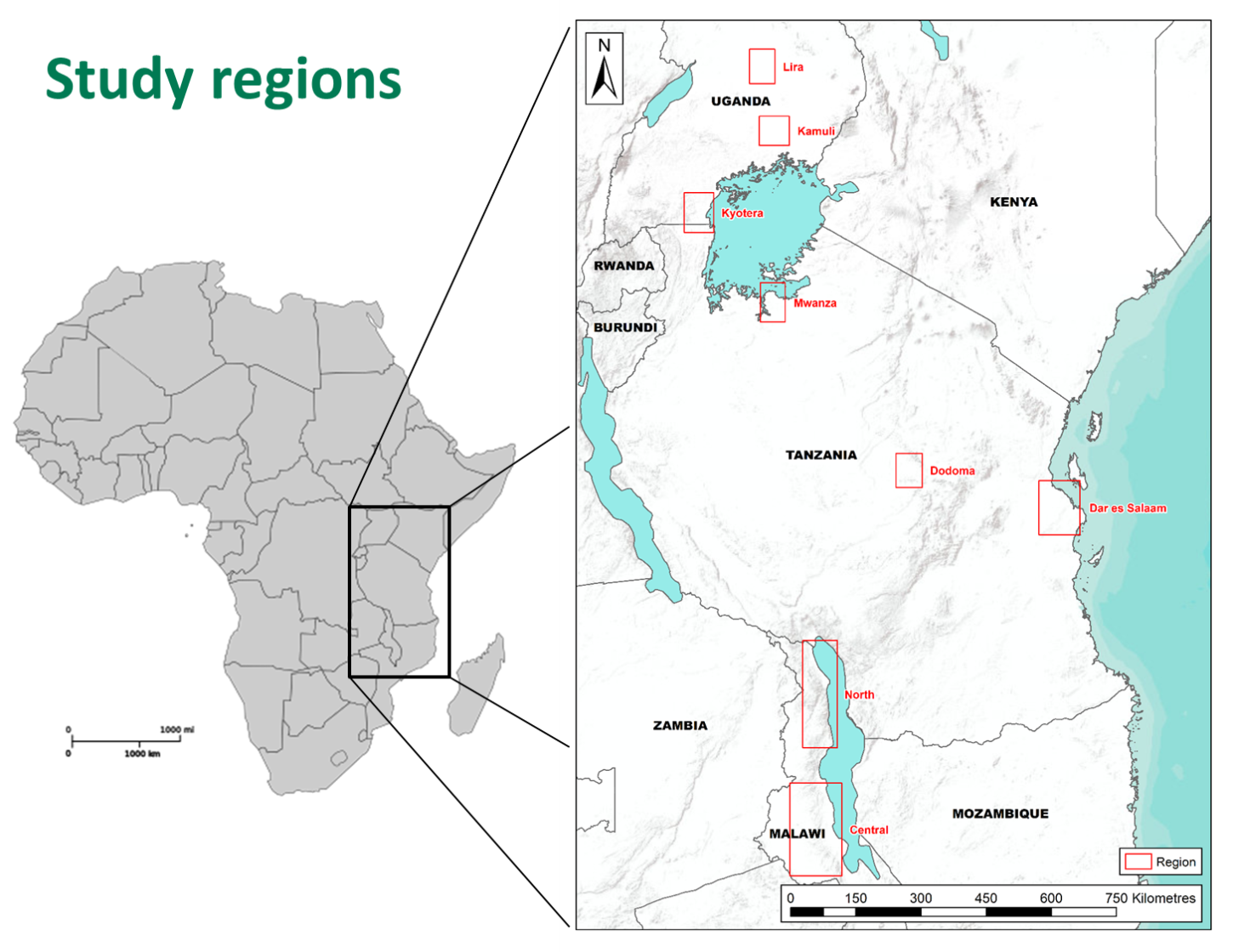


Figure S1: Map of the Sub-Saharan study region showing areas that were compared in terms of Cassava management regime in this study.

**
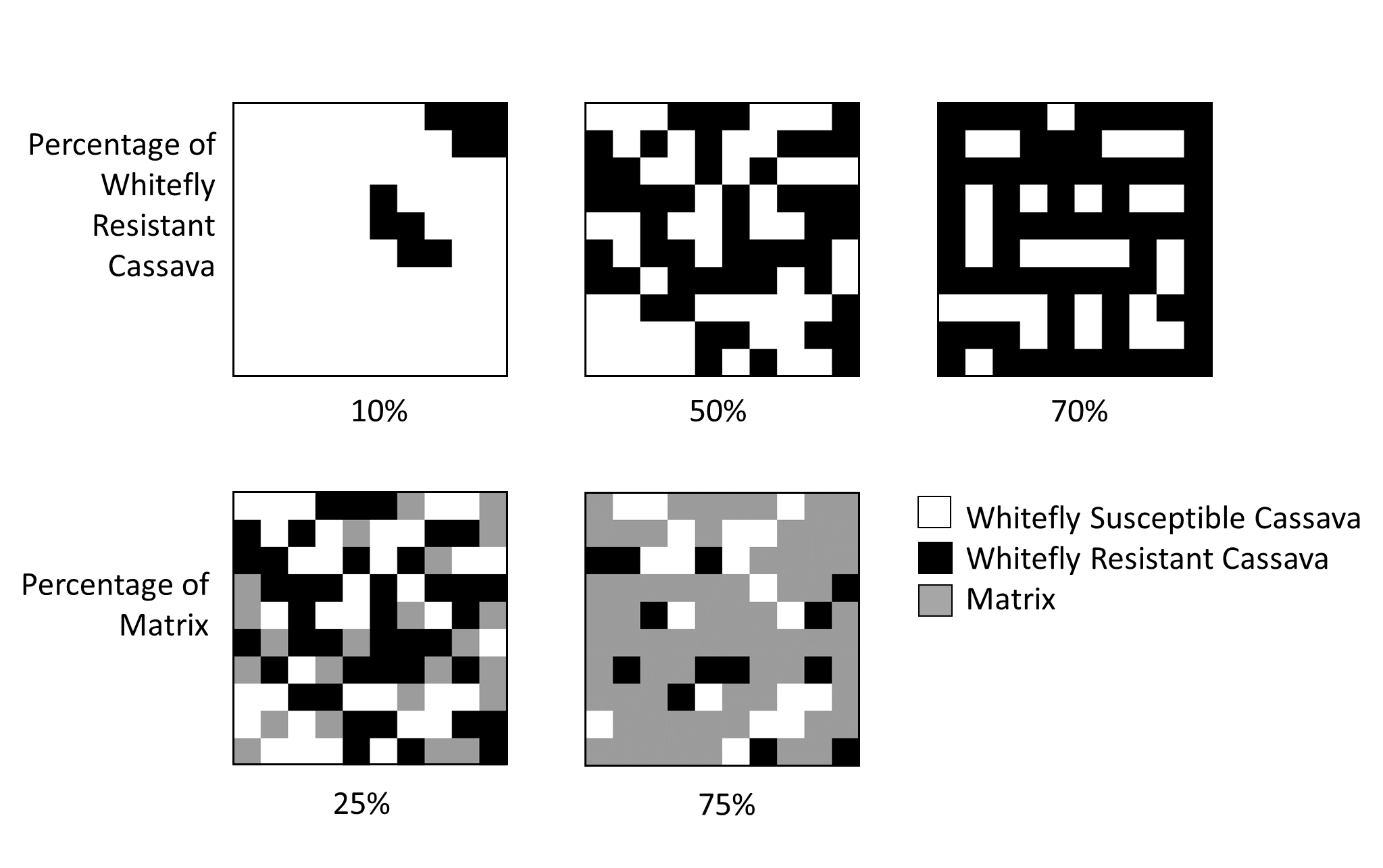
**

Figure S2: Artificial landscape scenarios used as model input in this study


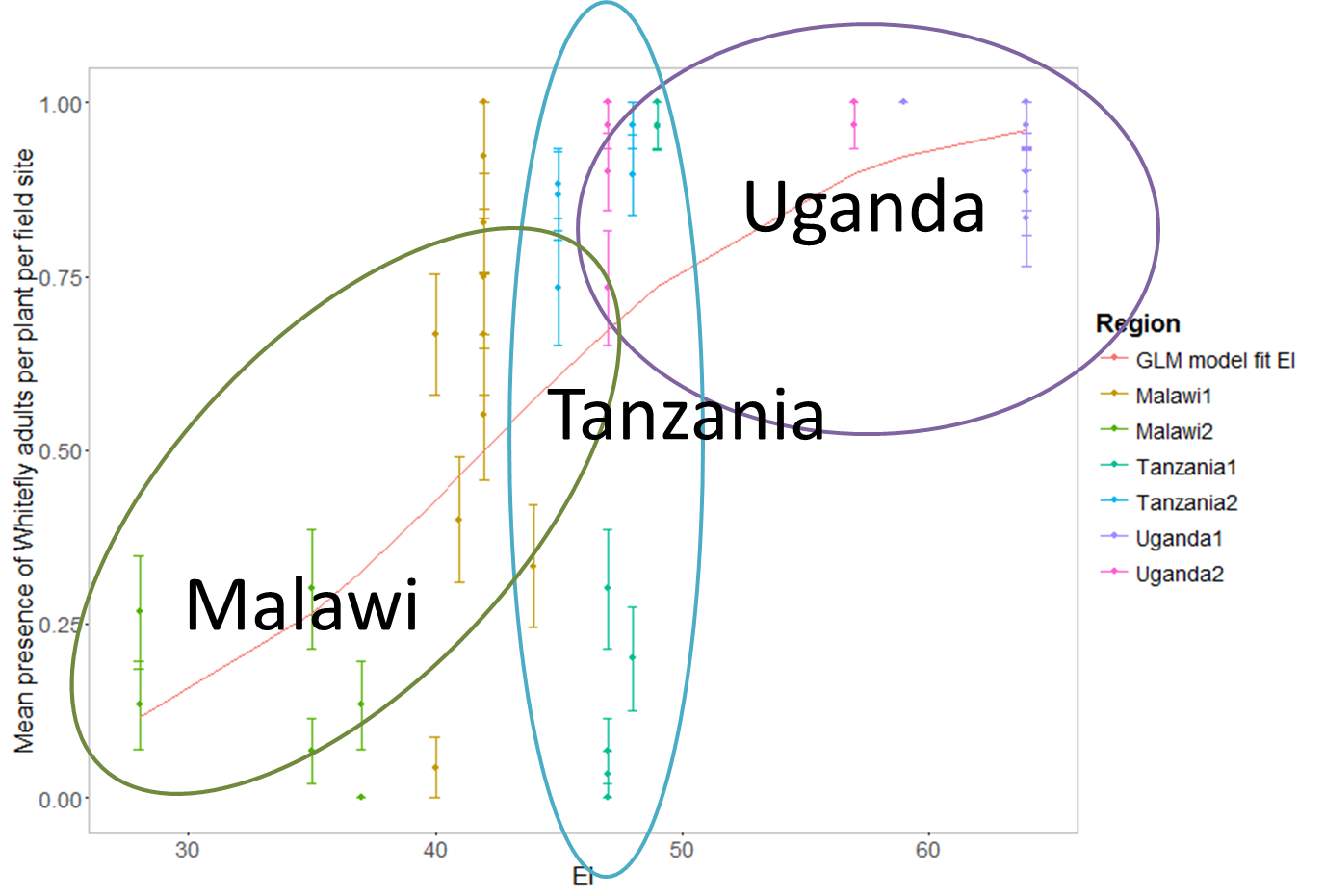


Figure S3: Observed presence of whitefly adults per plant vs the environmental Index (EI) calculated using a climatic niche model^30^
